# ADAM10 promotes cell growth, migration, and invasion in osteosarcoma via regulating E-cadherin/β-catenin signaling pathway and is regulated by miR-122-5p

**DOI:** 10.1186/s12935-020-01174-2

**Published:** 2020-03-30

**Authors:** Quan Yuan, Honghao Yu, Jianhua Chen, Xiaoyu Song, Li Sun

**Affiliations:** 1 Department of Orthopedics, Shengjing Hospital of China Medical University, 36 Sanhao Street, Heping District, Shenyang, 110004 People’s Republic of China; 2 Institute of Translational Medicine, China Medical University, Shenyang, 110122 People’s Republic of China; 3 Department of Nephrology, The First Hospital of China Medical University, Shenyang, 110001 People’s Republic of China

**Keywords:** Osteosarcoma, ADAM10, Cell growth, E-cadherin/β-catenin signaling pathway, MiR-122-5p

## Abstract

**Background:**

Osteosarcoma is a malignant bone tumor. Increasing evidences have revealed that a disintegrin and metalloproteinase 10 (ADAM10) is implicated in tumor development. The main purpose of this study is to explore the effects of ADAM10 on osteosarcoma cell functions and the underlying molecular mechanisms.

**Methods:**

Western blot and quantitative real-time PCR were performed to detect the expression of ADAM10 in one osteoblast (hFOB 1.19) and six osteosarcoma cells (Saos-2, SW1353, HOS, U-2OS, MG63, and 143B). The biological functions of ADAM10 in osteosarcoma cells were measured by cell counting kit-8 assay, flow cytometry, wound healing assay, and transwell assay. The interaction between miR-122-5p and ADAM10 was validated using dual-luciferase reporter assay. The effect of ADAM10 on the tumorigenicity of osteosarcoma cells was evaluated in a nude mice model in vivo.

**Results:**

We found that the expression of ADAM10 was relatively high in osteosarcoma cells compared with that in osteoblast. ADAM10 promoted osteosarcoma cell growth, migration, and invasion. Mechanism studies showed that knockdown of ADAM10 inactivated E-cadherin/β-catenin signaling pathway, as evidenced by increased the level of E-cadherin, reduced nuclear translocation of β-catenin, and decreased the levels of MMP-9, Cyclin D1, c-Myc, and Survivin. Downregulation of ADAM10 suppressed the tumorigenicity of osteosarcoma cells in vivo. Furthermore, ADAM10 was validated to be a downstream target of microRNA-122-5p (miR-122-5p). MiR-122-5p-induced inhibition of cell proliferation, migration, and invasion was reversed by overexpression of ADAM10 in osteosarcoma cells.

**Conclusions:**

Collectively, the key findings of this study are that ADAM10 promotes osteosarcoma cell proliferation, migration, and invasion by regulating E-cadherin/β-catenin signaling pathway, and miR-122-5p can target ADAM10, indicating that miR-122-5p/ADAM10 axis might serve as a therapeutic target of osteosarcoma.

## Background

Osteosarcoma, as the bone tumor, mainly affects children, adolescents, and young adults [1]. The introduction of multiagent chemotherapy has improved long-term survival rates from 20 to 65% in 5 years. Osteosarcoma has a strong ability to metastasize, and 85% of metastatic disease occurs in the lung, which is listed as one of the most common causes of cancer-related death. Thus, it was warranted to explore new therapeutic targets for improvement of the treatment and prognosis of patients with osteosarcoma.

A disintegrin and metalloproteases (ADAMs), a fascinating family of transmembrane and secreted proteins, are involved in “ectodomain shedding” of diverse growth factors, cytokines, receptors, and adhesion molecules [2]. ADAM family plays an important role in cell proliferation, migration, invasion, and angiogenesis, and it is also related to the metastasis of human tumors [3]. ADAM10, one of the numbers of ADAM family, can release extracellular regions of membrane-bound proteins [4]. The previous studies revealed that the expression of ADAM10 was overexpressed in a variety of tumors including liver cancer [5], melanoma [6], gastric cancer [7], lung cancer [8], pancreatic cancer [9], and bladder cancer [10]. In addition, the silencing of ADAM10 inhibited significantly cell growth and metastasis [11–13]. Thorsten Maretzky et al. [14] found that the shedding of E-cadherin caused by ADAM10 regulated the β-catenin subcellular localization and its downstream genes. Guo groups [15] found that ADAM10 could promote migration and invasion of human non-small cell lung cancer cells via activating Notch1 signaling pathway. Furthermore, ADAM10 was also regulated by a variety of upstream signaling indicators including SFRPs [16] and miR-122-5p [17]. Zhao et al. [18] indicated that the ADAM10 expression was significantly increased with the development of osteosarcoma progression, suggesting that ADAM10 may play an important role in the osteosarcoma progression. However, to the best of our known, the effects of ADAM10 on cell functions and the underlying molecular mechanisms in osteosarcoma have never been reported.

In this study, we find that ADAM10 can promote cell proliferation, migration, and invasion. Furthermore, we indicate that knockdown of ADAM10 can inactivate E-cadherin/β-catenin signaling pathway, and ADAM10 knockdown inhibit tumorigenicity of osteosarcoma cells in the hypodermis of nude mice. In addition, miR-122-5p can target ADAM10 in osteosarcoma cells.

## Methods

### Cell culture

Osteosarcoma cells Saos-2 (CL-0202), SW1353 (CL-0447), and HOS (iCell-h099) were purchased from Procell Company (Wuhan, China). U-2OS (ZQ0121), MG63 (ZQ0403), and 143B (ZQ0455) were purchased from Zhong Qiao Xin Zhou Biotechnology Company (Shanghai, China). Osteoblast hFOB 1.19 (ZQ0402) was purchased from Zhong Qiao Xin Zhou Biotechnology Company. All of the cell lines were authenticated by short tandem repeat DNA profiling and were found to be free of mycoplasma infection. Saos-2 cells were cultured in McCoy’s 5A medium (PM150710, Procell Company) with 15% fetal bovine serum (FBS) (P10033, Hyclone, USA). SW1353, U-2OS, and 143B cells were cultured in Dulbecco’s modified Eagle medium (DMEM) (12100-46, Gibco, USA) with 10% FBS. MG63 and HOS cells were cultured in minimum Eagle’s medium with 10% FBS, and hFOB 1.19 cells were cultured in DMEM/F12 medium with 10% FBS and 0.3 mg/ml G418. All cells were maintained in the humidified indicators at 37 °C with 5% CO_2_.

### Cell transfection

HOS and SW1353 cells were transiently transfected with pcDNA3.1–ADAM10 plasmids (Genscript, Nanjing, China) or their NCs using lipofectamine 2000 (11668-019, Invitrogen, USA). U-2OS and MG63 cells were transiently transfected with PRNAH1.1 plasmids (Genscript) containing ADAM10 shRNA or their NCs using lipofectamine 2000 (11668-019, Invitrogen). shRNA-1-ADAM10 sequences were GATCCGGGGTCTGTTATTGATGGAAGATTCAAGAGATCTTCCATCAATAACAGACCCTTTTTA; shRNA-2-ADAM10 sequences were GATCCGGGTCTCATGTACCTCCCAAAGTTCAAGAGACTTTGGGAGGTACATGAGACCTTTTTA; control shRNA sequences were GATCCCCTTCTCCGAACGTGTCACGTTTCAAGAGAACGTGACACGTTCGGAGAATTTTT.

### Western blot

Western blot assay was used to detect the protein expression [19]. Cells and tumor tissues were lysed using RIPA Lysis buffer (R0010, Solarbio, Beijing, China) to obtain total proteins. The cytoplasmic protein and nuclear protein were obtained using nuclear protein extraction kit (R0050, Solarbio) according to the manufacturer’s instructions. Protein concentration was assessed through BCA protein assay kit (PC0020, Solarbio). The proteins (40 μg/lane) were loaded and separated by sodium dodecyl sulfate polyacrylamide gel electrophoresis (SDS-PAGE). Separated protein bands were then transferred to polyvinylidene difluoride membranes (PVDF). The membranes were blocked with 5% nonfat milk and incubated with primary antibodies overnight at 4 °C. The primary antibodies used were as follows: ADAM10 (#14194, 1:500, CST, USA), E-cadherin (#610181, 1:500, BD Biosciences, USA), β-catenin antibody (#8480, 1:1000, CST), Matrix metalloprotein (MMP)-9 antibody (10375-2-AP, 1:1000, Proteintech, China), cyclinD1 antibody (#2922, 1:1000, CST), c-myc antibody (10828-1-AP, 1:1000, Proteintech), survivin antibody (10508-1-AP, 1:1000, Proteintech), GAPDH antibody (60004-1-Ig, 1:10000, Proteintech), and Histone H3 (GTX122148, 1:5000, Gene Tex, USA). Subsequently,the membranes were cultured with secondary antibodies including HRP conjugated goat anti-rabbit lgG (SE134, 1:3000, Solarbio) and HRP conjugated goat anti-mouse lgG (SE131, 1:3000, Solarbio) at room temperature for 30 min. Last, proteins were visualized using enhanced chemiluminescence reagent solution (PE0010, Solarbio) and relative protein levels were analyzed using Gel-Pro-Analyzer.

### Quantitative real-time PCR

The mRNA expression of ADAM10 was detected by Quantitative real-time PCR [20]. Total RNA was isolated from cells using total RNA extraction kit (DP419, Tiangen company, Beijing, China). The extracted RNAs were then reverse transcribed into cDNAs via M-MLV reverse transcriptase (NG212, Takara, Beijing, China). The primer information was as listed below: ADAM10-F:5′-TATTACGGAACACGAGAA-3′; ADAM10-R: 5′-AACGGAAAGGATTTGTAG-3′; GAPDH-F: 5′-GACCTGACCTGCCGTCTAG-3′; GAPDH-R: 5′-AGGAGTGGGTGTCGCTGT-3′. The reaction was carried out using an Exicycler 96 Bioneer (Bioneer Corporation, Daejeon, Korea). The relative expression levels of mRNA ADAM10 were analyzed using the 2^−△△CT^ method.

### Cell Counting Kit-8 (CCK-8) assay

Cell proliferation was analyzed by a CCK-8 kit (KGA317, Beyotime Institute of Biotechnology) [21]. Briefly, cells (4 × 10^3^ per well) were seeded into 96-well plates and each group contained five replicates. Twenty-four hours after cell transfection, 10 μl CCK-8 solutions were added to each well after 0, 24, 48, 72, and 96 h, respectively. The optical density (OD) was measured afterwards at 450 nm with a microplate reader (Biotek, Winooski, Vermont, USA).

### Flow cytometry

Annexin V-FITC apoptosis detection kit (C1062, Beyotime Institute of Biotechnology) was used to double stain cells through Annexin V-FITC and Propidium Iodide (PI) based on the manufacturer protocol, followed by fluorescence checking via NovoCyte Flow Cytometers (Acea Biosciences, CA, USA) [19]. Briefly, the cells were stained with 5 µL of Annexin V-FITC and 10 µL of PI (propidium iodide) to each tube. The cells were gently vortexed and incubated in the dark at room temperature for 15 min. The samples were analyzed by a flow cytometer.

### Wound healing assay

The cell migration was detected by wound healing assay [22]. The cells were seeded in 6-well plates. When the cells reached confluence, wounds were made by scratching with a 200  μL pipette tip. The scratch of each group was observed using an inverted phase contrast microscope at 0  h, 24  h, and 48  h.

### Transwell assay

The transwell assay was performed using corning transwell chambers (3422, Corning, New York, USA) [22]. The cells (1 × 10^4^) were seeded into the upper chamber, and the medium contained 30% FBS was added to the lower chamber. After 2 h incubation, the cells were fixed with 4% paraformaldehyde and stained with 0.4% crystal violet. The average number of invasion cells was used as a measure of invasion capacity.

### Elisa

E-cadherin concentrations were checked by ELISA kit (EK0561, Boster Biological Company, Wuhan, China) according to manufacturer protocol [14]. Briefly, the samples were washed and incubated with TMB substrate solution for 20 min at 37 °C in the dark. The color reaction was suspended by adding 100 μl TMB stop solution and the OD value was read at 450 nm in a microplate reader.

### Immunofluorescence assay

The expression and distribution of β-catenin were detected by immunofluorescence assay [23]. Cells: Cell slides were fixed using 4% paraformaldehyde for 15 min and incubated with 0.1% Triton X-100 (ST795, Beyotime Institute of Biotechnology). Cell slides were then blocked with goat serum for 15 min and incubated with β-catenin antibody (51067-2-AP, 1:200, Proteintech) overnight at 4 °C. After three times PBS washing, cell slides were incubated with Cy3-labelled secondary antibody (A0516, 1:200, Beyotime Institute of Biotechnology). The slices were incubated with DAPI and sealed with anti-fluorescent quenching agents (S2100, Solarbio). Typical images were captured under a microscope (× 400 magnification) (Olumpus, Japan). Tissues: The tumor tissues were dehydrated and put into xylene for 30 min. Tumor tissues were embedded in paraffin and sliced into 5 μm sections. Next, tissue section was immersed in xylene for dewaxing followed by incubating with Cy3-labelled secondary antibody (A0516, 1:200, Beyotime Institute of Biotechnology). Then tissue sections were stained with DAPI and sealed with anti-fluorescent quenching reagent (S2100, Solarbio). Typical images were captured under a microscope (×400 magnification) (Olumpus).

### Tumorigenesis in nude mice

A total of 24 nude mice were equally divided into 4 groups. For in vivo experiments, stable cell lines with knockdown of ADAM10 (U-2OS and MG-63) were screened with medium containing 500 μg/ml G418 (11811, Invitrogen) after transfection for 24 h with a similar fresh medium every 2 days. Stably transfected U-2OS cells or MG63 cells or their NC cells were then injected into subcutaneous tissue of 6 nude mice respectively. The tumor size was measured every 3 days after tumor formation (Day 7) through vernier caliper. Three weeks later, the mice were sacrificed and the tumors were removed for weighting and further detection [24]. This study was approved by the Institutional Animal Care and Use Committee of Shengjing Hospital of China Medical University and carried out according to the Guidelines for the Care and Use of Laboratory Animals.

### Dual-luciferase reporter assay

The wild-type (WT) or mutant (MT) 3′UTR sequence of ADAM10 containing predicted miR-122-5p binding site was inserted into pmirGLO vector. Then vectors and miR-122-5p mimic or its NC (GenePharma, Suzhou, China) were co-transfected into 293T cells (ZQ0033, Zhong Qiao Xin Zhou Biotechnology Company). The binding activity of miR-122-5p with ADAM10 was assessed by calculating the ratio of firefly luciferase to renilla luciferase activities.

### Statistical analysis

All data were analyzed using GraphPad Prism 7. The cell experiments were repeated at least three times and results were displayed as mean ± SD. Mean values were compared by One-way ANOVA, Two-way ANOVA and *T* test. A statistically significant difference in this study was adopted as *P *< 0.05.

## Results

### ADAM10 expressions were higher in the osteosarcoma cells

**Fig. 1:**
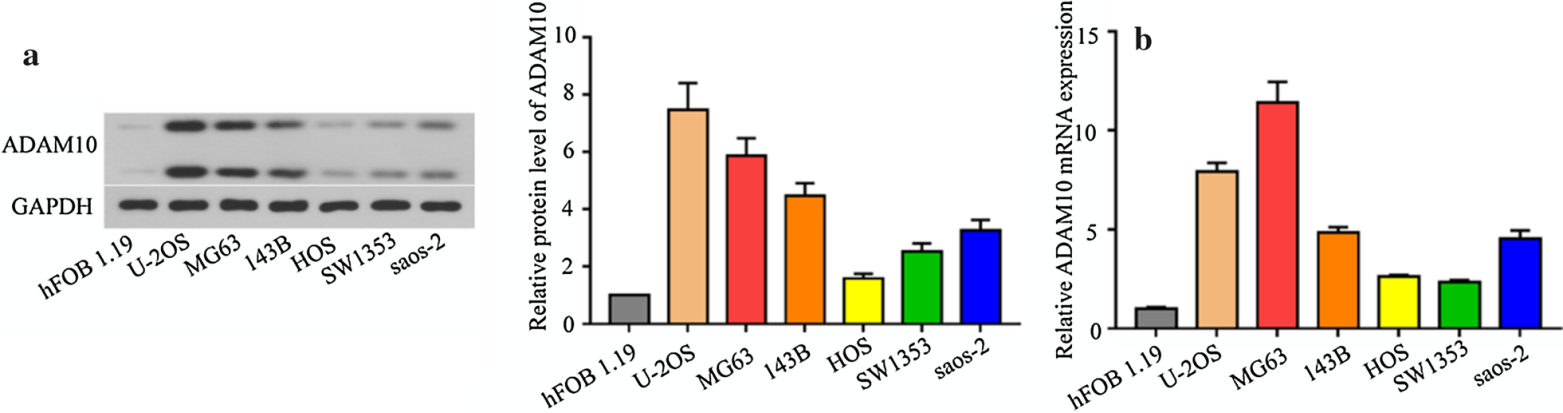
ADAM10 expression in human osteosarcoma cell lines and an osteoblast cell line. Western blot (**a**) and real-time PCR (**b**) analyzed the ADAM10 expression in osteoblast and osteosarcoma cells

We investigated the ADAM10 expression (active form and pro form) in one osteoblast (hFOB 1.19) and six osteosarcoma cells (Saos-2, SW1353, HOS, U-2OS, MG63, and 143B) through western blot and real-time PCR, respectively. Overall, the ADAM10 expression in six osteosarcoma cells was higher than that in the osteoblast hFOB1.19 at both protein levels (Fig. 1a) and mRNA levels (Fig. 1b). Furthermore, the ADAM10 expression was relatively lower in the HOS and SW1353 cells, but higher in the U-2OS and MG63 cells.

### ADAM10 overexpression increased osteosarcoma cell proliferation, migration, and invasion

**Fig. 2:**
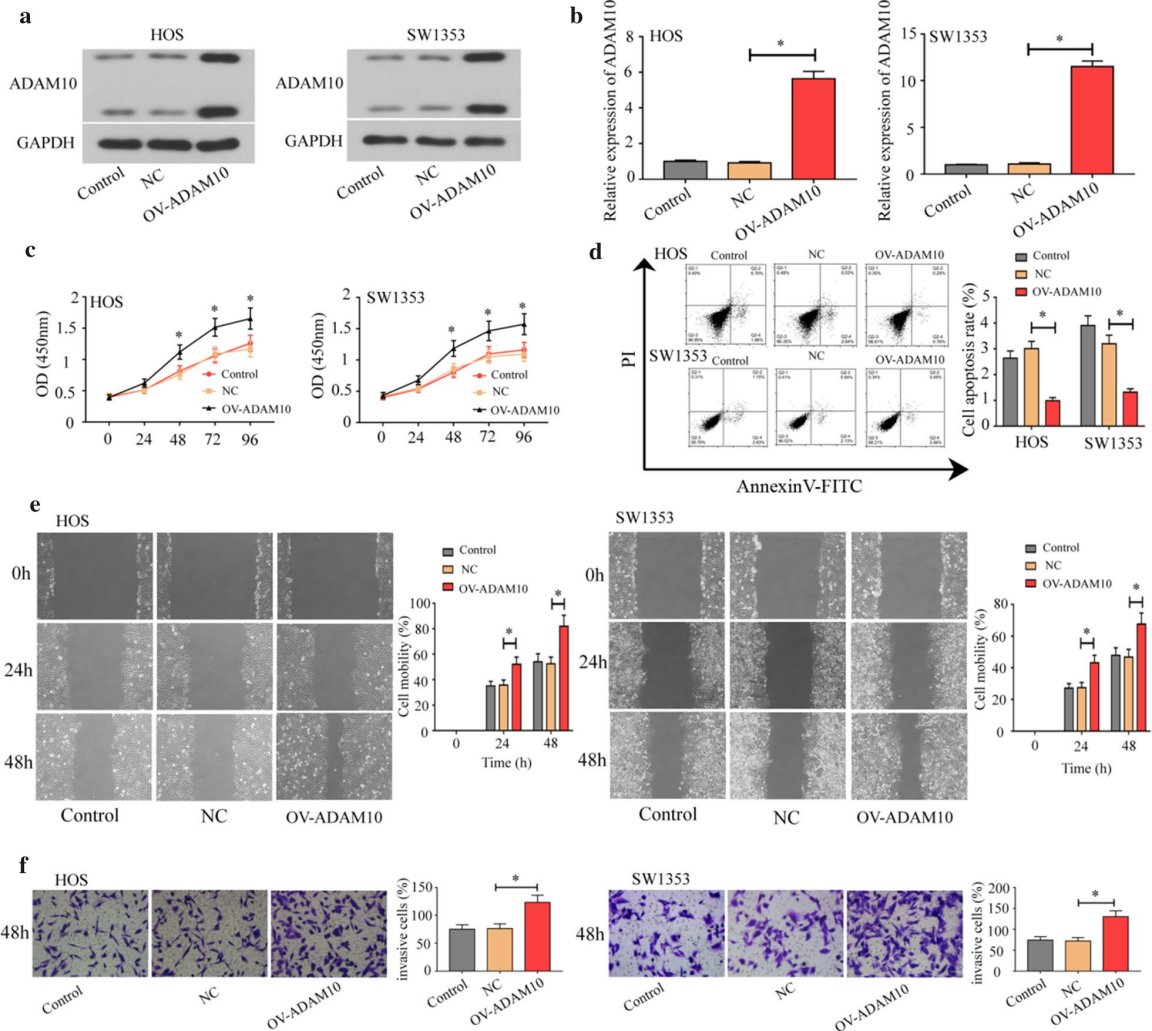
Overexpression of ADAM10 promoted cell growth, migration, and invasion in osteosarcoma cells (HOS and SW1353). Western blot (**a**) and real-time PCR (**b**) analyzed the ADAM10 expression in ADAM10-ovexpressing osteosarcoma cells. CCK-8 (**c**) was used to detect cell proliferation. Flow cytometer (**d**) was used to analyze cell apoptosis. Wound healing assay (**e**) was used to detect cell migration. Transwell assay was used to evaluate cell invasion (**f**) (*P < 0.05)

To have a better understanding of how ADAM10 affected osteosarcoma cell function, an ADAM10-overexpressing plasmid was transfected into two cells (HOS and SW1353) with relatively low ADAM10 expression. Figure 2a, b showed that ADAM10 expression was upregulated in the ADAM10-overexpressing cells. ADAM10 overexpression could promote cell proliferation (Fig. 2c). Figure 2d revealed a downward trend of cell apoptosis in ADAM10 overexpressing cells. Further, ADAM10 overexpression promoted cell migration (Fig. 2e) and invasion (Fig. 2f).

### ADAM10 knockdown decreased osteosarcoma cell proliferation, migration and invasion but increased cell apoptosis

**Fig. 3:**
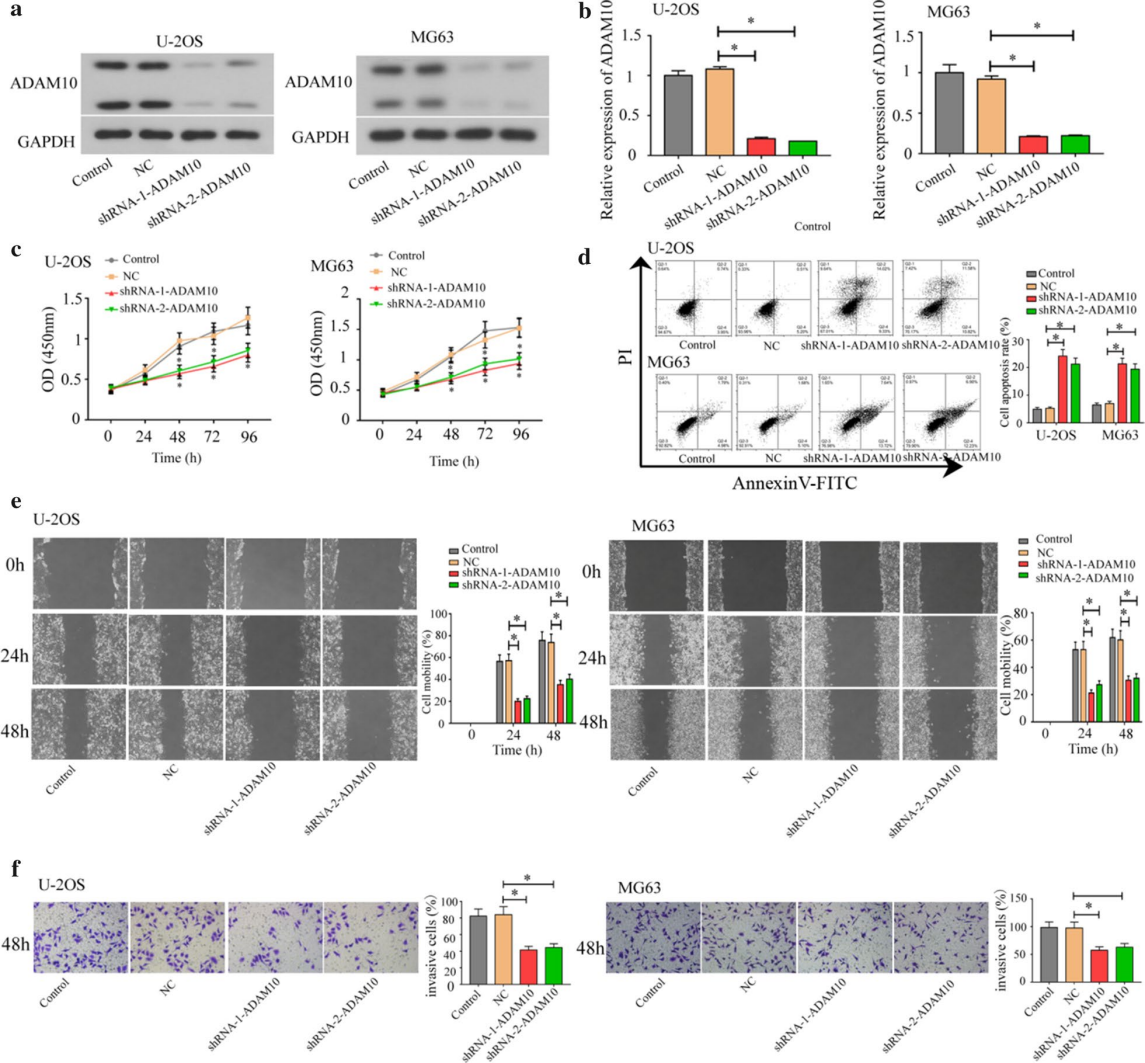
Knockdown of ADAM10 inhibited cell growth, migration, and invasion in osteosarcoma cells (U-2OS and MG63). Western blot (**a**) and real-time PCR (**b**) analyzed the ADAM10 expression in ADAM10-silenced osteosarcoma cells. CCK-8 (**c**) was used to evaluate the proliferation ability. Flow cytometer (**d**) was used to detect cell apoptosis. Wound healing assay (**e**) was used to detect cell migration. Transwell was used to analyze cell invasion (**f**) (*P < 0.05)

Meanwhile, the U-2OS and MG63 cells with higher ADAM10 expressions were adopted to do the transfection with two ADAM10 shRNAs to knock down its expressions. As shown in Fig. 3a, b, results of western blot and real-time PCR assays showed that ADAM10 expression was significantly decreased in ADAM10-silenced osteosarcoma cells. Knockdown of ADAM10 could inhibit cell proliferation (Fig. 3c) and promote cell apoptosis (Fig. 3d). Furthermore, knockdown of ADAM10 inhibited cell migration (Fig. 3e) and invasion (Fig. 3f).

### ADAM10 knockdown affected E-cadherin/β-catenin signaling pathway in the osteosarcoma cells

**Fig. 4:**
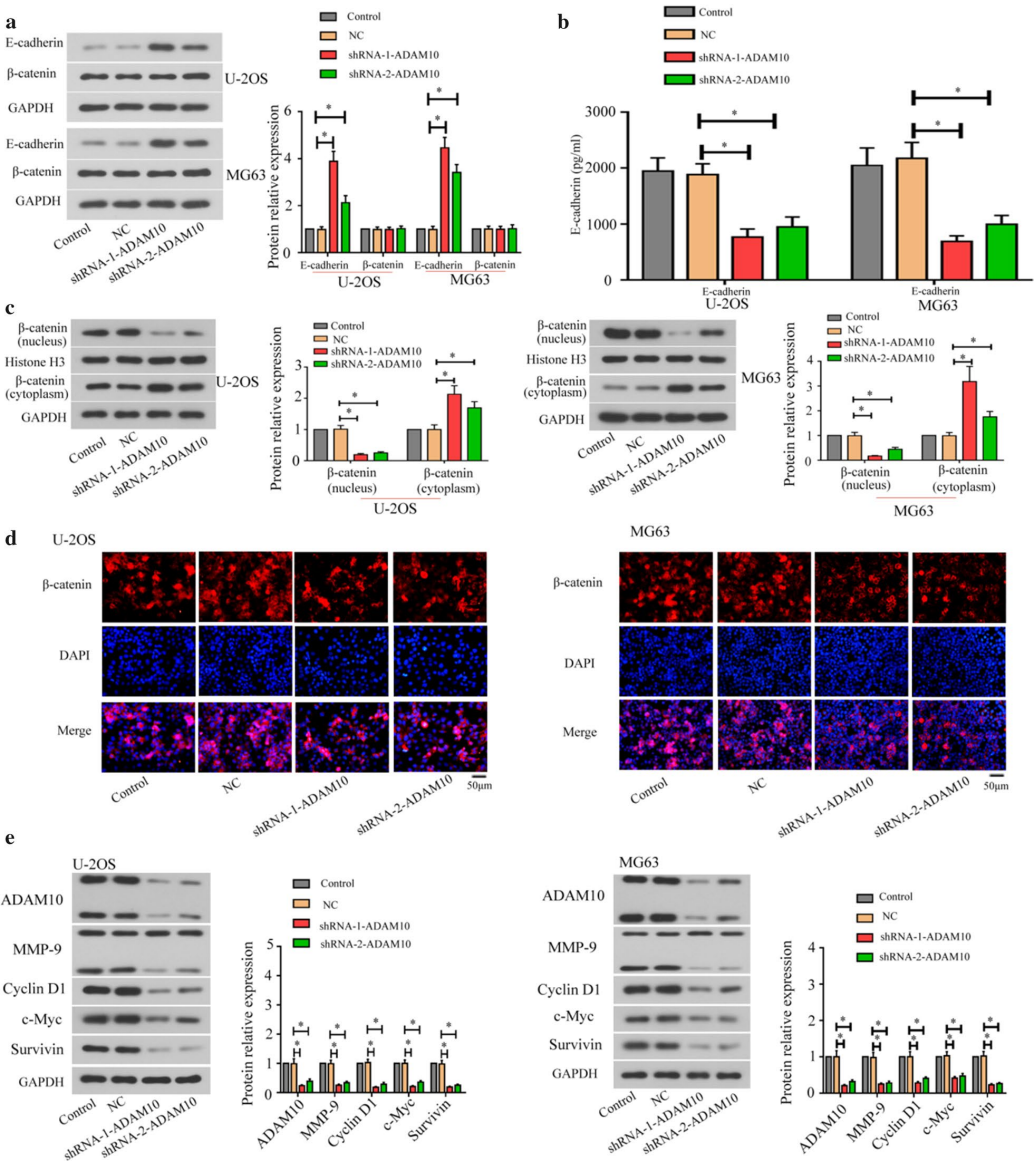
Knockdown of ADAM10 inactivated E-cadherin/β-catenin signaling pathway in osteosarcoma cells. Western blot (**a**) was used to analyze the expression of E-cadherin and β-catenin. ELISA (**b**) was used to evaluate the E-cadherin concentration. The β-catenin (nucleus and cytoplasm) expression was analyzed by western blot (**c**). The localization of β-catenin was detected by immunofluorescence assay (**d**). The expression of ADAM10, MMP-9, Cyclin D1, c-Myc, and Survivin was analyzed by western blot (**e**) (*P < 0.05)

In order to investigate the effects of ADAM10 knockdown on E-cadherin/β-catenin signaling pathway, the U-2OS and MG63 cells were transfected with ADAM10-shRNA for 48 h. Figure 4a showed that the expression of total E-cadherin was increased and the expression of total β-catenin did not change in the ADAM10-silenced cells. The decreased levels of soluble E-cadherin were found in supernatants of ADAM10-silenced U-2OS and MG63 cells as measured by ELISA with a soluble E-cadherin–specific antibody (Fig. 4b). These data suggested that ADAM10 induced E-cadherin ectodomain shedding, resulting in an increase of soluble E-cadherin. Furthermore, the protein expressions of β-catenin were detected in both nucleus and cytoplasm using western blot in the ADAM10-silenced cells. The results in both cell lines showed that knockdown of ADAM10 decreased the expression of β-catenin in the nuclear but increased the expression of β-catenin in the cytoplasm (Fig. 4c). Immunofluorescence assays showed that knockdown of ADAM10 inhibited the nuclear translocation of β-catenin (Fig. 4d). In addition, the levels of ADAM10, MMP-9, Cyclin D1, c-Myc, and Survivin were decreased in the ADAM10-silenced cells (Fig. 4e). This result demonstrates that ADAM10 down-regulation inhibits E-cadherin/β-catenin signaling pathway in osteosarcoma cells.

### ADAM10 knockdown inhibited tumorigenesis of osteosarcoma cells in vivo

**Fig. 5:**
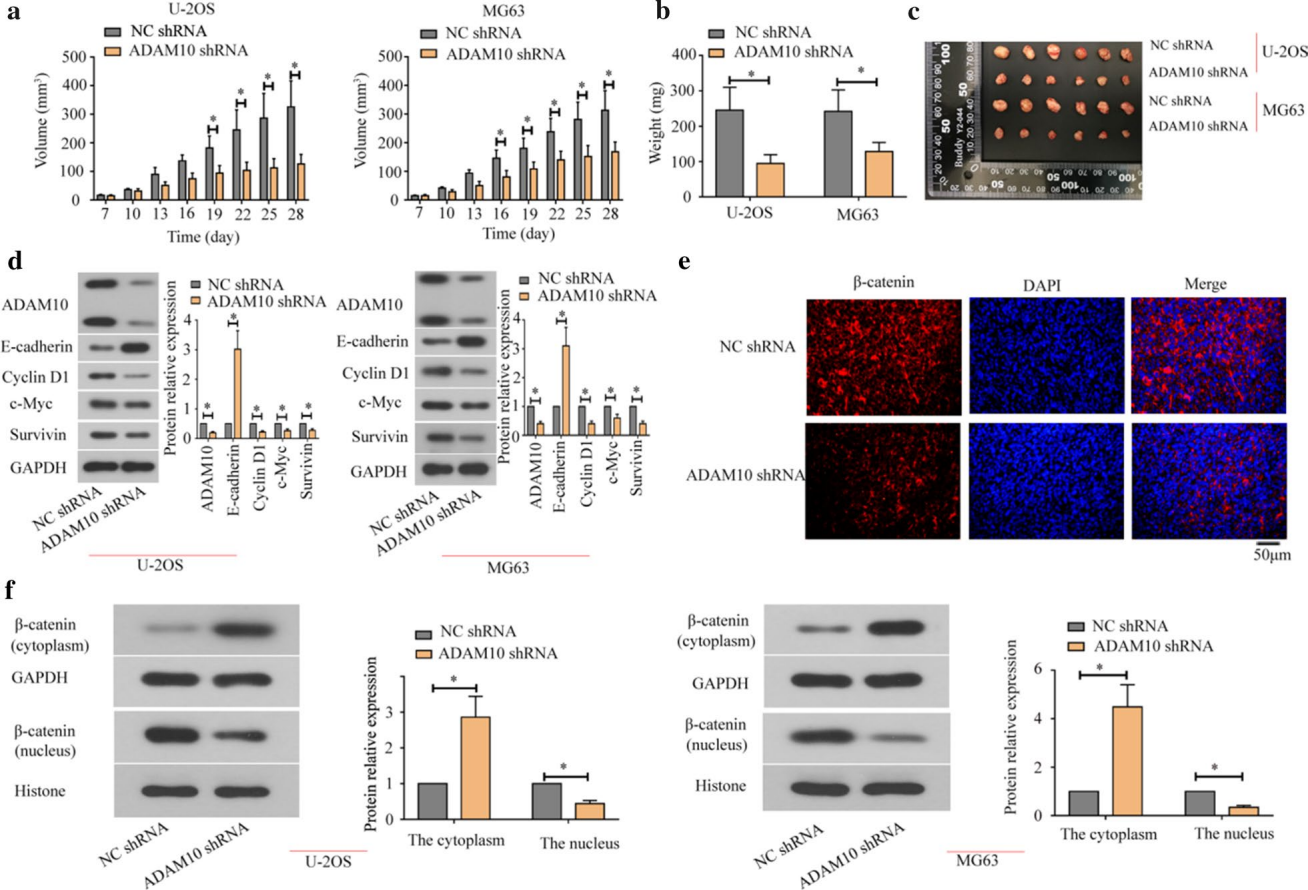
Knockdown of ADAM10 inhibited tumorigenicity of osteosarcoma cells. A single cell suspension of 0.5 mL containing 5 × 10^6^ of transfected cells was subcutaneously injected into each mouse at the left under-axillary. Tumor volume was measured every 3 days since day 7 after cell injection (**a**). Tumors were weight (**b**) and taken photo up 28 days after cell injection (**c**). The expressions of ADAM10, E-cadherin, Cyclin D1, c-Myc, and Survivin in osteosarcoma tissues were detected by western blot (**d**). The localization and expression of β-catenin were detected by immunofluorescence assay (**e**) and western blot (**f**) in the osteosarcoma tissues (*P < 0.05)

We established the stable transfected cell line (U-2OS and MG63) expressing low level of ADAM10. CCK-8 assay detected the proliferation of stably transfected cell lines, the result showed that knockdown of ADAM10 could inhibit cell proliferation (Additional file 1: Fig S1). Then ADAM10-silenced U-2OS and MG63 cells or their NC cells were injected into subcutaneous tissue of nude mice. We demonstrated that the volume and weight were decreased in the ADAM10-silenced osteosarcoma tissues (Fig. 5a, b), and ADAM10 knockdown inhibited tumorigenicity of osteosarcoma cells (Fig. 5c). Besides, western blot assays revealed that the expression of E-cadherin and β-catenin (cytoplasm) was increased, and the expression of ADAM10, Cyclin D1, c-Myc, Survivin, and β-catenin (nucleus) was reduced in the ADAM10-silenced tissues (Fig. 5d, f). Immunofluorescence assay showed that knockdown of ADAM10 inhibited the nuclear translocation of β-catenin (Fig. 5e). Therefore, our findings demonstrate that ADAM10 knockdown suppresses the tumor development and E-cadherin/β-catenin signaling pathway in osteosarcoma tissues.

### MiR-122-5p negatively regulated ADAM10 expressions directly in the osteosarcoma cells

**Fig. 6:**
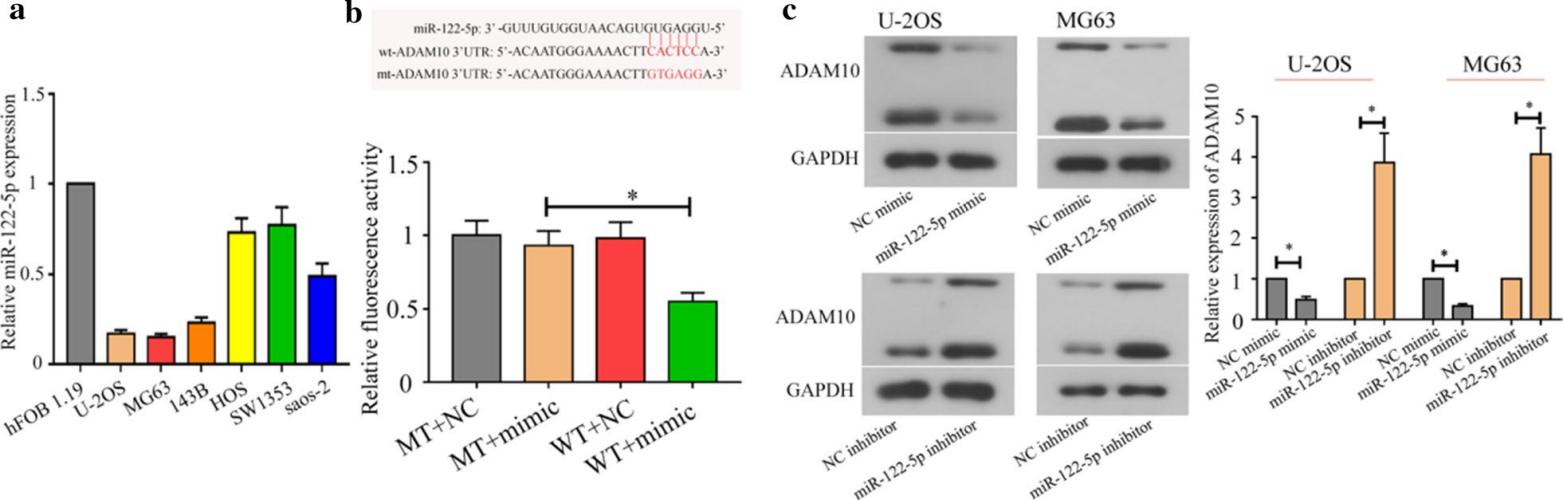
miR-122-5p could target ADAM10. Real-time PCR (**a**) analyzed the miR-122-5p expression in osteoblast and osteosarcoma cells. The targeting effect between miR-122-5p and ADAM10 was assessed using the dual-luciferase activity assay (**b**). Protein levels of ADAM10 in osteosarcoma cells after miR-122-5p mimic or miR-122-5p inhibitor transfection were evaluated by western blot assay (**c**) (*P < 0.05)

ADAM10 was predicted to be a downstream target gene of miR-122-5p. The miR-122-5p expression in six osteosarcoma cells was lower than that in the osteoblast hFOB1.19 (Fig. 6a). As shown in Fig. 6b, miR-122-5p mimics notably inhibited the luciferase activity in WT + mimic group compared to MT + mimic, indicating that miR-122-5p could bind with ADAM10. Furthermore, western blot assay showed that the expression of ADAM10 was significantly suppressed in U-2OS and MG63 cells after miR-122-5p mimic transfection (Fig. 6c). The results reveal that ADAM10 is a downstream target gene of miR-122-5p and miR-122-5p negatively regulated ADAM10 expressions directly in the osteosarcoma cells.

### MiR-122-5p affected osteosarcoma cell functions via regulating ADAM10

**Fig. 7:**
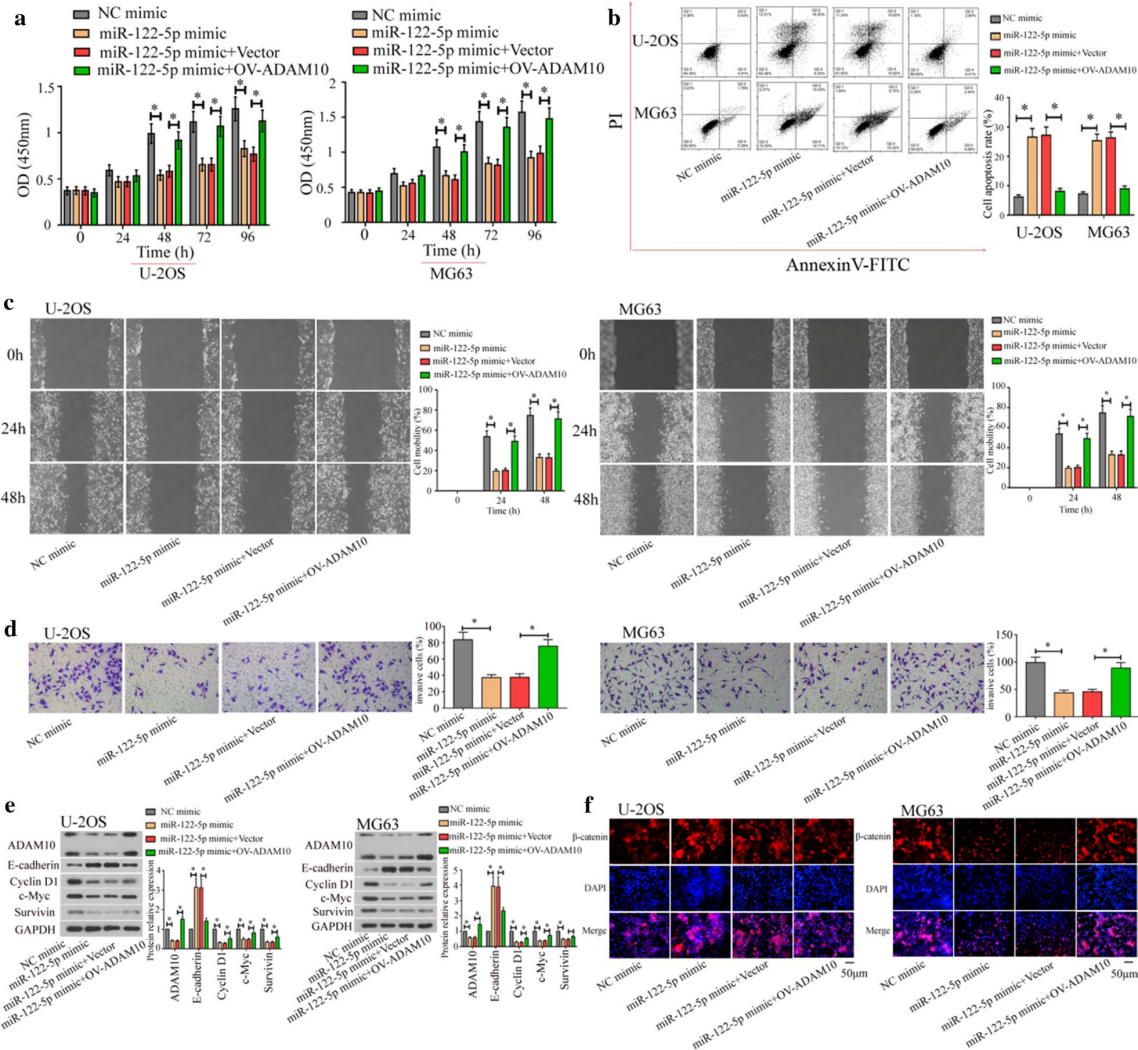
ADAM10 overexpression reversed the inhibition the effect of miR-122-5p on osteosarcoma cells. Cell proliferation was evaluated using CCK-8 assay (**a**). Cell apoptosis was detected by flow cytometer (**b**). Cell migration was performed by wound healing assay (**c**). Cell invasion was detected by transwell assay (**d**). The expression of ADAM10, MMP-9, Cyclin D1, c-Myc, and Survivin was analyzed by western blot (**e**). The localization and expression of β-catenin were detected by immunofluorescence assay (**f**) (*P < 0.05)

**Fig. 8:**
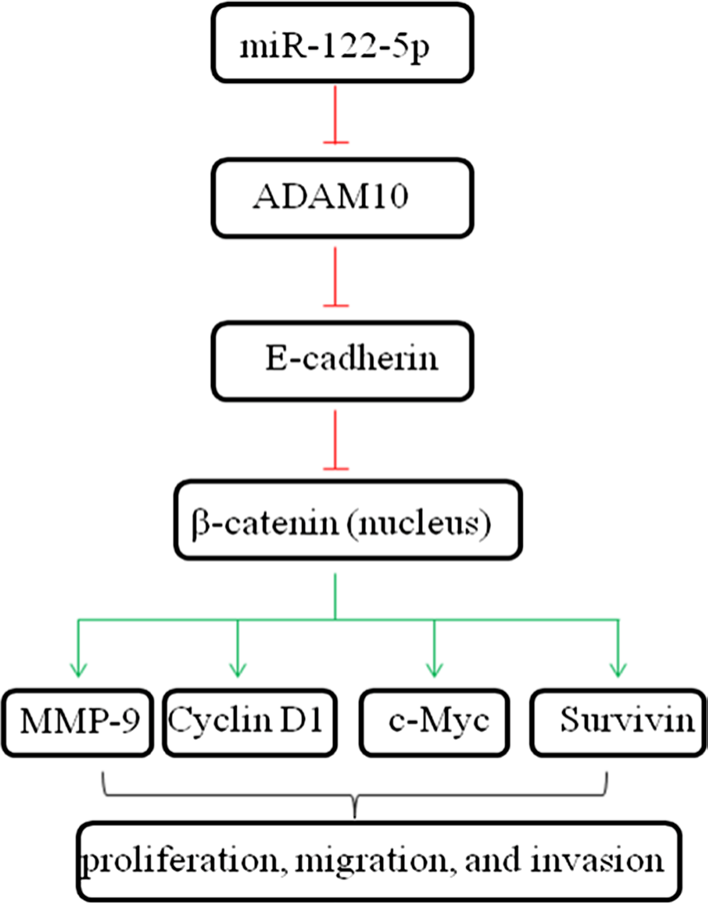
A schematic illustration of ADAM10 regulates E-cadherin/β-catenin signaling pathway which in turn is regulated by miR-122-5p

In the above results, we found that ADAM10 could promote osteosarcoma cell proliferation, migration, and, invasion via E-cadherin/β-catenin signaling pathway and miR-122-5p could directly target ADAM10. Furthermore, we explore whether miR-122-5p affected osteosarcoma cell functions by targeting ADAM10. MiR-122-5p mimic could inhibit cell proliferation (Fig. 7a), migration (Fig. 7c), and invasion (Fig. 7d), and promote cell apoptosis (Fig. 7b) in U-2OS and MG63 cells. In addition, miR-122-5p mimic increased the expression of E-cadherin and decreased the expression of ADAM10, Cyclin D1, c-Myc, and Survivin (Fig. 7e). Furthermore, miR-122-5p mimic inhibited the nuclear translocation of β-catenin (Fig. 7f). Those alterations caused by miR-122-5p mimic could be reversed by overexpression of ADAM10. Collectively, ADAM10 regulates E-cadherin/β-catenin signaling pathway which in turn is regulated by miR-122-5p. A schematic illustration was shown in Fig. 8.

## Discussion

Osteosarcoma is a highly malignant bone cancer. Despite many years of intensive laboratory and clinical research, the 5-year survival of patients with metastatic osteosarcoma is only 20% [25]. Therefore, understanding the osteosarcoma cell functions and underlying molecular mechanisms is important for formulating new therapeutic strategies. In this study, we focused on the roles of ADAM10 on the osteosarcoma cells and found that ADAM10 promoted osteosarcoma cell proliferation, migration, and invasion by regulating E-cadherin/β-catenin signaling pathway and ADAM10 knockdown inhibited tumorigenicity of osteosarcoma cells. In addition, miR-122-5p inhibited cell proliferation, migration, and invasion by targeting ADAM10.

ADAM10 regulated proliferation of bladder cancer cells [10]. Ko et al. [26] revealed that ADAM10 expression promoted cell growth of oral squamous cell carcinoma. Arima et al. [27] demonstrated that knockdown of ADAM10 expression decreased cell growth of prostate cancer. Studies found that ADAM10 promoted cell migration via regulating modulation of CD44 in the pituitary adenoma cell [28] and melanoma cell [29]. Guo et al. [13] found that ADAM10 was correlated with cell migration and invasion in human non-small cell lung cancer. In addition, the ADAM10 expression increased with the progression of osteosarcoma [16]. Based on these data, it is reasonable to speculate that ADAM10 may play a role in osteosarcoma cell growth, migration and invasion. And we also found that the expression of ADAM10 could promote cell proliferation, migration, and invasion of osteosarcoma.

ADAM10 could cleave E-cadherin, and the ectodomain shedding of E-cadherin is a unique way to control the biological function of E-cadherin [30]. β-catenin could dissociatd from the cytoplasmic tail of E-cadherin and its translocation into the nucleus to regulate the expression of certain genes [31]. In addition, thorsten et al. [12] found that ADAM10 mediated E-cadherin shedding and regualted β-catenin translocation. In aother study, ADAM10 decreased E-cadherin protein level and inhibited β-catenin pathway in hepatocellular carcinoma [32]. In the present study, we demonstrated that knockdown of ADAM10 could inhibit the E-cadherin/β-catenin signaling pathway. Furthermore, β-catenin could regulate its target genes including MMP-9, Cyclin D1, c-Myc, and Survivin [33–36]. Knockdown of ADAM10 decreased the levels of those target genes, also indicating that ADAM10 could regulate E-cadherin/β-catenin signaling pathway.

In addtion, we found that miR-122-5p could bind directly with ADAM10. Further rescue experiments revealed that ADAM10 overexpression could abrogate the miR-122-5p-mediated inhibition of cell proliferation, migration, and invasion in osteosarcoma cells. Studies reported that ADAM10 was targeted by other miRNAs. For instance, miR-449a inhibited cell invasion and migration by targeting ADAM10 in human non-small cell lung carcinoma [37]. Zhu et al. [38] found that miR-23a targeting ADAM10 contributed to epileptogenesis in temporal lobe epilepsy. Besides, miR-365 inhibited cell growth, migration, and invasion by binding to ADAM10 [39]. Therefore, there are maybe some other miRNAs targeting ADAM10 to regulate osteosarcoma progression, which need more explorations in the future.

## Conclusions

In summary, ADAM10 plays an important role to promote cell proliferation, migration, and invasion through an E-cadherin/β-catenin signaling pathway and miR-122-5p can be regarded as a negatively upstream regulator of ADAM10 directly to be involved in these regulations. Therefore, we deduce that miR-122-5p/ADAM10 axis may also serve as a therapeutic target for osteosarcoma treatment.

## Supplementary information

**Additional file 1: Fig S1.** Cells proliferation was detected by CCK-8 assay in the stably transfected cell lines (*P < 0.05).

## Data Availability

The datasets supporting the conclusions of the current study are available from the corresponding author on reasonable request. Please contact corresponding author, if you want to request the dataset.
