# Supplementary figures and images for: ADAM10 promotes cell growth, migration, and invasion in osteosarcoma via regulating E-cadherin/β-catenin signaling pathway and is regulated by miR-122-5p
Source: Cancer Cell Int. 2020 Mar 30;20:99. doi: 10.1186/s12935-020-01174-2 (PMC7106760; doi:10.1186/s12935-020-01174-2)

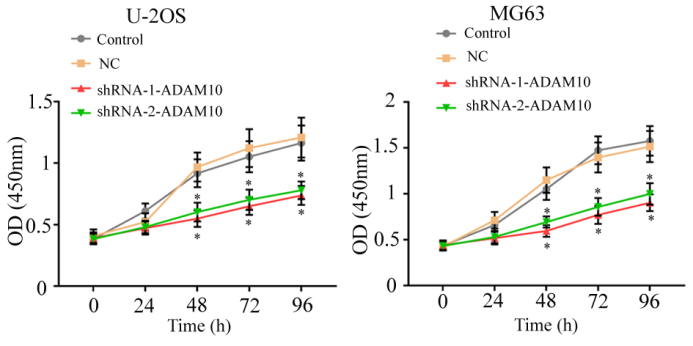


**Fig S1**

Supplement: Supplementary file 1 — Additional file 1: Fig S1. Cells proliferation was detected by CCK-8 assay in the stably transfected cell lines (*P < 0.05). [file 12935_2020_1174_MOESM1_ESM.docx]
